# Rabbit Dental Abnormalities: Investigation of Conformational Risk Factors in a Pedigree Rabbit Population

**DOI:** 10.3390/ani15070980

**Published:** 2025-03-28

**Authors:** Maria A. Jackson, Michaela Betts, Joanna Hedley, Charlotte C. Burn

**Affiliations:** 1 Department of Pathobiology and Population Sciences, The Royal Veterinary College, Hawkshead Lane, Hatfield AL9 7TA, UK; mbetts3@rvc.ac.uk (M.B.); cburn@rvc.ac.uk (C.C.B.); 2 Beaumont Sainsbury Animal Hospital, The Royal Veterinary College, Royal College Street, London NW1 0TU, UK; jhedley@rvc.ac.uk

**Keywords:** animal welfare, conformation, *Oryctolagus cuniculus*, risk factors, selective breeding, teeth

## Abstract

Lop-eared and brachycephalic conformations have inconsistently been associated with dental abnormalities in pet and rescue rabbit populations. We investigated conformational risk factors for dental abnormalities in a pedigree population where conformation could be confirmed. Otoscopic oral examination was performed on breeder-volunteered pedigree rabbits at British Rabbit Council shows and studs. Multivariable models were used for risk factor analysis of 14 possible dental disease signs. In 435 rabbits examined, 68.28% showed no incisor abnormalities and 55.40% showed no cheek teeth abnormalities. Lop-eared rabbits had higher odds than erect-eared rabbits for ocular discharge (odds ratio [OR]: 4.034, *p* = 0.007) but not for any dental abnormalities. Erect-eared rabbits showed higher odds of reacting to incisor examination than lop-eared rabbits (OR: 1.666, *p* = 0.003). Brachycephaly showed no associations with dental abnormalities, whilst more dolichocephalic rabbits had increased odds of cheek teeth step or wave mouth (OR: 1.394, *p* = 0.004). Other conformational and signalment-related risk factor results were mixed. Brachycephaly showed no significant predisposition to dental abnormalities, and the clinical relevance of predisposition to ocular discharge in lop-eared rabbits is unclear. Conformation appears less important than husbandry factors for preventing rabbit dental disease. Generalising the results to companion rabbits is not advised due to husbandry and population differences.

## Simple Summary

Rabbits with lop ears and flattened head shapes have sometimes been found to have a higher chance of developing dental disease than those with erect ears and longer faces, like those of wild rabbits. We visually examined the mouths of 435 pedigree rabbits at British Rabbit Council shows and studs, whilst recording rabbits’ ear types and head shapes, to assess if any tooth abnormalities were linked to ear type and head shape. Many rabbits had no incisor abnormalities (68.28%) and no cheek teeth abnormalities (55.40%). Lop ears were not a significant risk factor for any dental abnormalities but were associated with ocular discharge. Erect-eared rabbits reacted more frequently to incisor examination. Flatter head shapes were not significantly associated with dental abnormalities, but those with longer head shapes had cheek ‘step or wave’ mouth more commonly. Ear type and head shape may be less important than husbandry for preventing rabbit dental disease. Some of the findings may not apply to pet rabbits because of husbandry and population differences.

## 1. Introduction

Dental disease is a common welfare issue in domestic rabbits, with recent studies estimating the prevalence to be 15.36% and 18.23% in UK pet rabbits [1,2]. Whilst several signalment-related and environmental risk factors are reported to be associated with the disease [1,2,3,4,5,6], some dental abnormalities have been suggested to be conformational. Rabbits with lop ears or brachycephalic head shapes may be predisposed to dental abnormalities [4,7], suggesting that it may be possible to reduce dental disease incidence and associated suffering by avoiding breeding rabbits of these conformations. If so, working with rabbit breeders could thus be an effective route to reducing the prevalence of rabbit dental disease. However, evidence for conformational predisposition to dental disease is currently inconsistent and requires further investigation before breeders can be advised on which, if any, conformations offer the greatest chance of good dental health in rabbits [1,2,4,7].

The study of risk factors for rabbit dental disease is important for animal welfare because the associated pain can be chronic and severe [8,9,10,11]. Dental disease is often a hidden illness that breeders and owners may be completely unaware of in their rabbits [3,5,12]. Likewise, in the early stages of disease progression, diagnosis can only be made using specialist veterinary equipment or imaging, especially for cheek teeth disease [13,14,15,16,17,18,19,20,21,22,23,24].

### 1.1. Associations Between Conformation and Dental Disease

No significant associations between any type of dental disease and ear conformation, head shape, or body weight were found in two large UK retrospective studies assessing clinical records of over 160,000 rabbits [1,2]. Lop-eared rabbits had shorter lifespans, but their causes of death did not differ significantly from erect-eared rabbits, suggesting dental disease was not implicated in that association [2]. However, there may have been some ear and head conformation misclassifications in those studies because these conformations were necessarily estimated from the reported breed in the veterinary record, rather than being directly observed. A smaller cross-sectional study involving oral otoscopic examination of 15 lop-eared and 15 erect-eared rabbits at a UK rescue centre reported that lop-eared rabbits had 23.3 times higher odds of incisor pathology, 12.0 times higher odds of molar overgrowth, and were significantly more likely to have molar spurs than erect-eared rabbits [7]. This study was, however, limited by a relatively small sample size from a single rescue centre, where rabbits theoretically could have been relinquished due to pre-existing dental disease. To our knowledge, no other empirical studies have reported statistically significant associations between ear conformation and dental disease to date.

Brachycephaly has been proposed as a risk factor for rabbit dental disease [15,25,26,27], perhaps partly because of its association with dental disease in cats [28,29] and dogs [30,31]. In a retrospective Thai study of 100 seemingly healthy rabbits and 100 rabbits with acquired dental disease, brachycephalic rabbits had 3.19 times higher odds of dental disease compared to rabbits with longer head shapes [4]. In that study, head shape was also designated based on recorded breed, and analysis was at only the univariable level, but acquired dental disease was diagnosed through physical examinations in all cases, and radiography in 75% of the cases. That study, however, contrasts with the lack of a significant association between head shape and dental disease presence found at multivariable level in the two large retrospective UK studies [1,2].

Dwarfism has also been suggested to be associated with congenital incisor malocclusion [14,15,25,27,32] and acquired incisor malocclusion secondary to an overall reduction in jaw size [14]. The autosomal recessive trait of hereditary maxillary brachygnathism reduces the skull and maxillary diastema length in rabbits leading to congenital incisor malocclusion [33], but its direct effect on acquired dental abnormalities remains unstudied. Three studies that systematically investigated whether smaller rabbit breeds are at greater risk of any dental disease than larger rabbit breeds have found no significant association [1,2,3]. An additional study that surveyed the husbandry of 102 pet rabbits reported that Dwarf Lops were significantly more likely to have dental issues than any other breed at a univariate analysis level, but this association disappeared when accounting for diet and age in multivariable analysis [5]. Another study of 1254 rabbit clinical records found no relationship between dwarfism and needing dental treatment at univariate level [34].

Additional, non-conformational risk factors for development of dental disease include male sex [1,2,3,4], old age [1,3,4,5,6], a diet comprising low volumes of hay [3,4], feeding muesli [5], and housing in a cage compared to systems that allowed free-ranging [3].

### 1.2. Aims, Scope, and Hypotheses

In this study, we aimed to further investigate conformational risk factors for dental abnormalities in a population of rabbits owned by breeders; ear conditions were also investigated as part of the same project, as described elsewhere [35].

The research reported here did not primarily aim to include formal clinical diagnosis of dental disease, but was carried out to generate evidence that could help guide breeding and selection of rabbits for good dental health and thus better associated welfare. Both early and subtle signs of potential dental disease were therefore included, even if their current clinical relevance was uncertain. Rabbit dental disease can have primary causes (e.g., hereditary maxillary brachygnathism [24,33,36]) and/or secondary causes (e.g., inadequate diet leading to progressive syndrome of acquired dental disease [PSADD] [8,16,25,27,32,34]). However, clinical signs of primary and secondary dental disease can overlap and have not been distinguished from each other, so all potential signs of both disease types were recorded in this study.

We used direct observation of conformational features and dental abnormalities in pedigree companion rabbits owned by British Rabbit Council (BRC) members. The BRC aims to ‘promote and continually discover rabbit welfare standards’ that must be upheld by their members and to ‘promote healthy and ethical breeding practices’ [37]. The BRC holds rabbit shows, or exhibitions, where rabbits of multiple recognised pedigree breeds are exhibited and judged in accordance with the relevant BRC breed standard. These breed standards describe the appearance and conformation that defines each of the breeds currently recognised [38]. The breed standards also define disqualifications to encourage good health; of particular relevance to the current study is that dental malocclusion or deformity is defined as a disqualification from showing for all BRC breeds, and BRC judges are trained to inspect the incisors and disqualify rabbits from winning at exhibitions on this basis [38]. Thus, this BRC study population had great potential relevance for using the study findings to make evidence-based recommendations for breeding rabbits with good dental health and consequent welfare.

The hypothesis was that, if lop-eared, brachycephalic, or dwarf rabbit conformations cause harmful developmental malformations of the mandibles or dental alignment, then—in multivariable analyses—rabbits of one or more of these conformations would consistently have higher odds of dental abnormalities compared with erect-eared, normocephalic (mesocephalic or dolichocephalic), or larger rabbits, respectively.

## 2. Materials and Methods

### 2.1. Study Design

The study population comprised pedigree rabbits belonging to BRC-registered breeders. Breeders were recruited through the membership magazine ‘Fur & Feather’, online BRC social media groups, and in-person during attendance of BRC rabbit shows. Breeders were informed of the aim of the study and participation was entirely voluntary, with the data being recorded in an anonymous format and written consent obtained from each breeder. Ethical approval for the study was given by the Royal Veterinary College Social Science Ethical Review Board (reference SR2023-0115).

Calculation of the minimum sample size required for the dental aspect of the study estimated that at least 108 rabbits (54 brachycephalic, 54 longer faced) would be required, based on Siriporn and Weerakhun (2014) [4] as a study that found a statistically significant effect of skull conformation on dental disease. For the calculation, we thus used a predicted 35% of longer faced rabbits having dental disease versus 63% of brachycephalic rabbits [4], with an 80% power to detect a two-tailed effect with 95% confidence, and the assumption of equal group sizes. As a more conservative estimate, based on the UK prevalence of rabbit dental disease diagnoses in first opinion practices being approximately 15% [1], and aiming to detect an odds ratio of 2.0, then a minimum of 432 rabbits would be required (216 of each conformation).

The inclusion criteria were any pedigree rabbits amenable to handling (without excessive fear or stress) belonging to, or with permission from, BRC-registered breeders over the age of 18 years old. Amenability to handling was based on breeder perception of the temperament of the rabbit and the behaviour of the rabbit during judging where applicable. The Polish rabbit breed was excluded from physical examination due to its very small body size combined with reported behaviourally reactive temperament leading to animal welfare concerns [38]. Unweaned, pregnant, or nursing rabbits were excluded from physical examination. For ethical reasons, rabbits displaying signs of pain or fear during the initial stages of examination, beyond the normal resistance expected of well-handled rabbits, and as defined by an ethogram (Supplementary Materials, Table S1), were also excluded.

### 2.2. Physical Examination

Two examiners, a veterinary surgeon and registered veterinary nurse with rabbit handling experience, were trained by two veterinary surgeons, including an exotics specialist, in the appropriate use of an otoscope for dental examination and appropriate categorisation of observations. The categorisations were not clinical diagnoses, and instead descriptions of dental and associated features that could range from mild divergences from the norm to possible clinically relevant signs.

An examination protocol and physical examination checklist were developed, and pilot tested 32 times over a period of six weeks from August 2023 to September 2023 at two different rescue centres, one veterinary practice, one university animal research unit, and two companion rabbit set-ups. The examination checklist included potential external signs of dental disease and categories for dental abnormalities within the mouth. Categorisation of each individual dental abnormality was discussed at length and refined in accordance with relevant literature and clinical expertise to most accurately quantify signs observed until a verbal agreement was reached between each researcher and the veterinary surgeons involved in the training process. The two training veterinary surgeons acted as independent assessors and were involved in ensuring the clinical assessment technique and grading system were used consistently by the examiners. The descriptive criteria for each variable investigated and the scoring system is defined in Supplementary Materials, Table S2.

Data were collected between 1 October 2023 and 8 February 2024 at BRC rabbit shows and studs of BRC-registered rabbit breeders in England and Scotland. The order of examination at shows was intended to be randomised, but this was not feasible because only certain rabbits were available at any given time due to the tight exhibition schedules. Rabbits of consenting owners were brought to a non-slip examination table by BRC members and show stewards. One examiner then gently restrained the rabbit against the table in a rabbit’s normal standing or seated position without restraint aids [39], whilst the other examiner performed the examination. The examination and recording of findings lasted between 3.5 and 10 min depending on the reactivity of the rabbit, although examination was terminated early if signs of pain, fear, or stress beyond normal resistance were displayed as described previously. The examiners alternated between examination and restraint of rabbits after every third rabbit, other than at one large show where the provision of an additional otoscope allowed both examiners to simultaneously examine rabbits.

**Figure 1:**
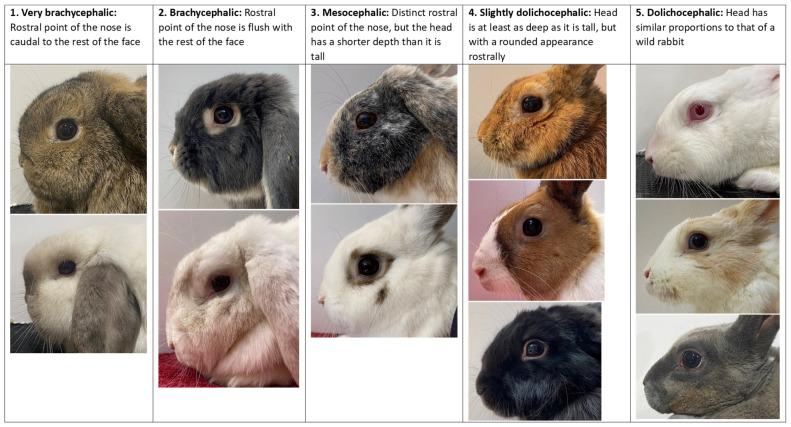
Photographic head shape scale for categorisation of rabbit head shapes from 1 (very brachycephalic) to 5 (dolichocephalic) during examination. Photographs taken by the authors.

Data were collected about each rabbit before physical examination including breed, sex, neuter status, BRC age bracket (under 5 months, 5 months and over), year of birth, and where available, exact date of birth. To preserve anonymity, breeder identities were not recorded. For rabbits where an exact date of birth was not available, the birth date was defined as the 1st July of the respective year of birth, reflecting the later end of the usual breeding schedule of BRC-registered rabbits [40]. Ring numbers (located on metal rings secured to a hind leg of BRC-registered rabbits for identification) were recorded to prevent re-examination of the same rabbit at shows. Breed-estimated bodyweight was defined as the mean of the weight range specified for an adult rabbit of each breed in the BRC standards, or by any single value provided where a range was not given [38]. Ear type (erect, lop) and fur length (very shorthair, shorthair, medium hair, long or semi-longhair) were determined from the recorded breed. Head shape was assessed independently by the examiner and categorised using a one to five (very brachycephalic to dolichocephalic) photographic scale created during the pilot phase (Figure 1).

An initial external visual assessment was then performed for the presence or absence of facial asymmetry, head tilt, fur wetness, any type of ocular discharge, and exophthalmos. The mandible and maxilla were also palpated for any abnormalities. Following external assessment, the ears were examined as described elsewhere [35]. Dental examination was then performed by first visually assessing the upper (maxillary) and lower (mandibular) incisors for abnormalities including malformations (any, missing teeth, fracture, ribbing), length (short, normal, long), colour (on a 0–2 scale), occlusal surface abnormalities (any, rough, slanted), and malocclusion (any, misdirection, maxillary brachygnathism, maxillary prognathism, incisors touching). Any absence of peg teeth was noted, but no other peg teeth abnormalities could be assessed due to difficulty visualising peg teeth in a conscious rabbit.

An otoscope with a metal cone (Heine BETA, Heine, Gilching, Germany) was then inserted into the diastema of the mouth bilaterally, beginning on the left side of the rabbit’s mouth, and orientated to visualise the ipsilateral row of cheek teeth (premolars and molars) and soft tissue surfaces initially, before re-orientation over the tongue to visualise the contralateral cheek teeth arcades. The process was then repeated for the right side of the mouth. Intra-oral examination was used to assess for cheek teeth malformation (any, missing teeth, fractures), tooth colour (on a 0–2 scale), tooth length (short, normal, long), step or wave mouth, spurs, and sharp edges (which were not deemed to be an abnormality). The first, rostral-most cheek tooth was recorded separately from the other, caudal cheek teeth in all four quadrants to prevent loss of data in reactive rabbits where only the first cheek tooth could be assessed. The tongue and oral mucosa were also examined for erythema, bleeding, laceration, ulceration, purulent discharge, and hyperplasia. Measures of reactivity to incisor and intra-oral examination were recorded, as defined by the ethogram. The results were recorded on an assessment checklist with space for free-text comments (Supplementary Materials, Table S3). Breeders were informed if any of their rabbits had dental abnormalities that warranted veterinary intervention.

Otoscope cones were cleaned between rabbits using paper towel and wipes to remove organic material, and disinfected using F10 disinfectant (Meadow’s Animal Healthcare, Loughborough, UK). A dilution of 1:100 and contact time of 15 min was applied as recommended by the manufacturer to ensure virucidal activity against common and potentially fatal caliciviruses (such as rabbit haemorrhagic disease) and enveloped viruses (such as myxomatosis) [41,42,43,44]. Otoscope cones were subsequently wiped with water wipes (WaterWipes, Drogheda, Ireland) to minimise disinfectant residue.

To test inter-observer reliability, where possible, every 6th rabbit was physically examined by both examiners. The show environment was often busy, and rabbits did not always tolerate examination by both examiners, so, dual observation was predominantly performed at usually quieter stud visits. Rabbits were examined by the initial examiner, and these results were used for the main dataset. Rabbits were then immediately afterwards examined by the second examiner purely for the purpose of testing inter-observer agreement. If the rabbit was displaying negative responses to examinations as defined by the ethogram, then the next amenable rabbit was selected instead.

### 2.3. Statistical Analysis

Data were cleaned in Microsoft Excel V.2411, and any duplicate rabbits had their second examination record removed. Upper and lower incisors, and upper and lower, left and right, and first and caudal cheek teeth were grouped together to facilitate statistical analysis and avoid multiple testing of the same factors. If only the upper incisors were examinable and the lower not, or vice versa, then the incisor examination was classed as not possible for that rabbit. The same was true for upper and lower, right and left, and first and caudal cheek teeth. The only exception was the cheek teeth length outcome variable, where first and caudal cheek teeth were kept separate. The tooth colour scale variable was converted to a binary (normal, abnormal) outcome due to rarity, as were tongue and oral mucosa lesions (converted to a single oral mucosa lesion variable).

Measures of reactivity to incisor and intra-oral examination were both converted to composite reactivity scores whereby each individual measure of reactivity contributed a value of 1 and added together per rabbit if multiple measures were recorded; rabbits who were unexaminable were automatically scored as the highest reactivity score plus 1.

IBM SPSS Statistics V.28 was used for all statistical analyses. Inter-observer agreement between the two examiners was assessed by using Cohen’s Kappa testing for binary categorical data and Kendall’s Coefficient of Concordance for ordinal data or scales. Agreement coefficients of 0.4 or lower were considered Poor [45]. To assess whether any disagreement was caused by differences between the order in which rabbits were examined (e.g., if rabbits consistently resisted the first examination either more, or less, than the second), the first and second observations for variables attaining Poor agreement were compared using a McNemar test for binary variables or Wilcoxon Signed Rank test for ordinal variables.

Binary logistic generalised estimating equations were used to assess potential risk factors for binary outcome variables that showed sufficient variation for analysis (at least 5% of rabbits being in the minority category). The composite reactivity score outcomes were tested with linear generalised estimating equation models, and residuals were visually checked for normalcy. Due to rarity, incisor reactivity was analysed with a binary logistic model for the presence or absence of any reactivity (any reactivity to incisor examination), and a linear model only for those rabbits who did display reactivity, with zero-scores removed (increasing reactivity to incisor examination); intra-oral reactivity was assessed separately as a single linear outcome (any and increasing reactivity to intra-oral examination). The predictors were rabbit age, sex, breed-estimated bodyweight, ear type, head shape, fur length, examiner, examination number, and examination location at show or stud. Breed was included as a random effect to account for the clustered structure of the data, with the working correlation matrix set to exchangeable. Missing values were excluded from models.

Ear type and head shape were forced into multivariable models regardless of association at univariable level as variables of a priori interest, and sex and age were included as both have been shown to influence dental disease [1,3,4,5]. Other risk factors with liberal associations in univariable modelling were taken forward to multivariable evaluation, as defined by *p* < 0.20. Model fit of alternative models was assessed using the corrected quasi likelihood under independence model criterion (QICC) number and the Wald Chi-square, where relevant. Multicollinearity was assessed via inflation of standard errors. Because older rabbits were mostly seen at studs, there was collinearity between age and examination location (show or stud), so these variables could not always be included in the same model. Age was the more biologically relevant predictor, so the models not including examination location were selected as the final models if the QICC was lower without this predictor.

In total, 14/47 outcomes showed sufficient variation for statistical analysis, generating 101 *p*-values where Type I errors (falsely significant findings occurring by chance) could have occurred [46]. Therefore, the false discovery rate (FDR) was corrected for [47], and the statistical significance level was set at *p* < 0.019. For completeness, results with *p*-values between the FDR-corrected threshold and the usual *p* < 0.05 significance threshold are reported as nonsignificant trends.

## 3. Results

### 3.1. Population Descriptives

Four-hundred and thirty-five rabbits representing 49 breeds were examined across eight shows (*n* = 272, 62.53%) and nine studs (*n* = 163, 37.47%). The most frequently examined breeds were Miniature Lop (*n* = 71, 16.32%), Netherland Dwarf (*n* = 55, 12.64%), and Miniature Rex (*n* = 41, 9.43%) (Table 1). Most rabbits were male (*n* = 275, 63.22%), of entire neuter status (*n* = 428, 98.39%), with erect ears (*n* = 266, 61.15%), and a brachycephalic head shape (*n* = 180, 41.38%). The median rabbit age was 1.29 years (interquartile range [IQR] 0.79 to 2.85), and median adult bodyweight was 2.16 kg (IQR 1.55 to 3.40). The two examiners examined 222 (51.03%) and 213 (48.97%) rabbits, respectively.

### 3.2. Inter-Observer Agreement

Observer agreement for the head shape scale from Very brachycephalic to Dolichocephalic was Almost perfect (95%; Kendall’s W = 0.970; Table 2). Most (32/47; 68.09%) outcome variables had insufficient variation to enable inter-observer agreement testing. The majority of the incisor abnormalities, cheek teeth length, and oral mucosa variables showed Excellent or Substantial agreement, as did any and increasing reactivity to intra-oral examination. In contrast, ocular discharge showed the poorest agreement. Unhealthy, slanted or curved incisor occlusal surfaces and any cheek teeth abnormality also showed Poor agreement. Comparisons of the first and second observations showed no significant order effects for dental observations, so this did not explain observer disagreements (e.g., rabbits were not consistently more reactive on the first or second examination).

### 3.3. General Clinical Signs of Disease

General clinical signs indicating possible dental abnormalities were mostly rare in the study population (Table 3). Thirteen rabbits (2.99%) displayed wet fur; five had wet fur on the chin, four around the nares, and four between or around the eyes. No rabbits were observed to have facial asymmetry or exophthalmos, but 146 rabbits (33.56%) were noted to have ocular discharge. One rabbit (0.23%) displayed a head tilt, three (0.69%) had abnormal maxillae, and one (0.23%) had an abnormal mandible.

### 3.4. Reactivity to Dental Examination

Three-hundred and forty rabbits (78.16%) did not display any reactivity to examination of the incisors, and the incisors of nine (1.84%) were completely unexaminable due to reactivity considered to be beyond the normal resistance expected (Table 4). Of the 95 rabbits (21.84%) who displayed reactivity to incisor examination, the most common behaviour was moving the head, and the median reactivity composite score was 1 with an IQR of 1–2.

All except six rabbits reacted in some way to intra-oral examination, and 55 (12.64%) were unexaminable at this point (Table 4). Of the 429 rabbits (98.62%) who displayed reactivity to intra-oral examination, the most common reaction was chewing the otoscope head, and the median reactivity composite score was 1 (IQR 1–3).

### 3.5. Dental Abnormalities

A total of 12 rabbits (2.76%) would not tolerate full examination of all incisors—three of which displayed reactivity that meant only their upper incisors could be examined—whilst 423 (97.24%) of rabbits did tolerate full incisor examination (Table 5). Incisor abnormalities were recorded in 126 rabbits (28.97% of all 435 rabbits). The most common abnormality was slanted or curved incisor occlusal surfaces in 111 rabbits (25.52%). Other incisor abnormalities were relatively rare. No rabbits had maxillary brachygnathism (underbite) or mandibular brachygnathism (overbite), but one (0.23%) had incisors that touched each other, and four (0.92%) showed misdirected incisors. Three rabbits (0.69%) had incisor fractures; free-text comments indicated that two of these had small fractures on the incisor tips (one of whom was known to chew the hutch chicken wire), whilst the other rabbit had known dental disease with multiple dental abnormalities, and the breeder mentioned they had used nail clippers to trim the incisors recently.

All cheek teeth were able to be examined in 379 rabbits (87.13%), and 138 rabbits (31.72% of all 435 rabbits) showed at least one dental abnormality, not including normal sharp cheek teeth edges (Table 6). The most common abnormalities were step or wave mouth in 54 rabbits (12.41%), and the first cheek tooth either being relatively long (*n* = 70, 16.09%) or short (*n* = 72, 16.55%). Spurs were rare (*n* = 15, 3.45%) whilst normal sharp edges were common (*n* = 323, 74.25%). In free-text comments, three of the four rabbits with fractured cheek teeth were described to have multiple dental abnormalities coinciding with intermediate to late-stage PSADD (including overgrown and missing cheek teeth, and gum hyperplasia), whilst the remaining rabbit had a chipped lingual aspect of one tooth. The three rabbits with missing cheek teeth were also all described to have multiple dental abnormalities, and two of the three rabbits with missing cheek teeth also all had fractured cheek teeth.

Most rabbits tolerated examination of the oral mucosa (*n* = 381, 87.59%) and 25 had evidence of any type of oral lesion (5.75% of the total 435 rabbits; Table 7). Eight of the sixteen rabbits with oral hyperaemia had relevant free-text comments recorded; five described hyperaemia in relation to overgrown cheek teeth, sharpness, or spurring, two noted unexplained pinprick red marks on the oral mucosa, and one described unexplained bruising on the inner lip. All eight rabbits with oral bleeding had comments recorded. One rabbit unfortunately experienced iatrogenic oral bleeding caused by the otoscope cone during a sudden head movement. Two rabbits experienced bleeding in relation to spurring and dental pathology, one appeared related to hay lodged between the cheek teeth and gums, and four were unexplained. The oral lacerations (*n* = 3) were unexplained, and the two rabbits with hyperplasia had multiple dental abnormalities and missing cheek teeth, one also with ulceration and bleeding.

### 3.6. Results of Multivariable Analysis

Only 11 dental abnormality outcomes were sufficiently common for analysis out of 44 recorded, but all three measures of reactivity (incisor reactivity presence, incisor reactivity score if present, and intra-oral reactivity score) were tested (Table 8). Lop-eared rabbits had approximately four times higher odds than erect-eared rabbits for having ocular discharge (*p* = 0.007), whilst, to a lesser extent, erect-eared rabbits had higher odds of displaying any reactivity to incisor examination than lop-eared rabbits (*p* = 0.003). More dolichocephalic rabbits had increased odds of cheek teeth step or wave mouth (*p* = 0.004), and rabbits with a more brachycephalic head shape showed no statistically significant predisposition to any dental abnormalities. As breed-estimated bodyweight increased, the odds of having ocular discharge increased (*p* < 0.001), as did the odds and scores of behavioural reactivity to incisor and intra-oral examination. Conversely, as breed-estimated bodyweight increased, the odds decreased for having cheek teeth sharp edges (*p* < 0.001).

Turning to signalment-related risk factors, male rabbits had approximately double the odds than females for having any incisor abnormality, including a slanted or curved incisor occlusal surface (*p* = 0.006) (Table 8). Males also had higher scores for any and increasing levels of reactivity to intra-oral examination (*p* = 0.003). Females had approximately double the odds than males for long first cheek teeth (*p* = 0.002). As age increased, the odds increased for ocular discharge (*p* = 0.006), any cheek teeth abnormality (*p* < 0.001), and cheek teeth step or wave mouth (*p* < 0.001). Younger rabbits had higher scores for increasing reactivity to incisor examination, but this was no longer significant following FDR correction. Fur length had inconsistent results across many dental variables with effects in most directions, so full results are reported in Supplementary Materials, Tables S4 and S5.

Regarding methodological factors, examination order was significant at the FDR adjusted level in five of the eight dental abnormality models where this variable was included, and none of the three models assessing reactivity to dental examination (Supplementary Materials, Tables S4 and S5). The effect of the examiner was significant in three of the four dental abnormality models where this variable was included, and in two of the three behavioural reactivity models. The effect of examination location at show or stud was not tested in any of the final dental abnormality models but was significant in all three behavioural reactivity models tested. The full results for generalised estimating equation models with all predictor variables can be seen in Supplementary Materials, Tables S4 and S5.

## 4. Discussion

This study aimed to further understanding of conformational predispositions to dental abnormalities, particularly in lop-eared, brachycephalic, or dwarf rabbits, via systematic observation of a non-clinical pedigree population. To achieve this, we obtained a volunteered sample of 435 rabbits representing 49 breeds, and most rabbits tolerated dental examination. Many dental abnormalities were uncommon, but 28.97% of rabbits showed incisor abnormalities, and 31.72% showed cheek teeth abnormalities. Conformation showed no consistent associations with dental abnormalities, with most dental abnormalities being statistically non-significant. Some of these abnormalities also had unknown clinical relevance. The findings of the present study, therefore, reiterate the multifactorial nature of dental abnormalities [1,3,48,49].

First, we discuss the conformational and signalment-related risk factors suggested by the results. The results that were not significant following FDR correction are not discussed as they may be type I errors and not reflect true risk factors for dental disease. We summarise by discussing the study limitations and overall implications.

### 4.1. Conformational Risk Factors for Dental Abnormalities

Our results did not support the hypothesis that any of the conformations tested here are consistently associated with dental disease risk. Only one variable—ocular discharge—was significantly increased in lop-eared rabbits, but its relation to dental disease in this study is uncertain as it was simply recorded as present or absent. Rabbit dental disease can present with persistent ocular discharge from occlusion of the nasolacrimal duct by overgrown tooth roots [50,51,52,53], but minor ocular discharge such as transient excessive tearing (e.g., hyperlacrimation or epiphora) can also result from temporary ocular irritants such as dust and hay [54,55]. Here, ocular discharge also attained Poor inter-observer agreement, with a strong and significant difference in the likelihood that the two researchers observed it in the sample of 20 rabbits. Binary outcome variables are known to lead to poor inter-observer agreement [45], as was the case for this variable, so distinguishing between mild and severe ocular discharge or detailed definitions of alternative types of discharge would be important in future research. A questionnaire of 548 rabbit owners worldwide did also identify brachycephalic rabbits had 1.852 times higher odds of having dacryocystitis than mesocephalic rabbits; however, owner reported cases could not be verified to distinguish the true presence of dacryocystitis from other less clinically significant forms of ocular discharge [56]. Thus, any association between ocular discharge and rabbit ear conformation requires verification through further research, and until then, this finding should not be relied upon.

The hypothesis that brachycephalic rabbits would have higher odds of dental abnormalities than mesocephalic or dolichocephalic rabbits, as initially detected in one smaller univariable study [4], was also not supported by these data. No dental abnormalities were associated with a shorter, more brachycephalic head shape. In fact, the odds of having cheek teeth step or wave mouth increased as head shapes lengthened and became more dolichocephalic. It is, however, unclear why more dolichocephalic rabbits would have increased odds of this dental abnormality. Further confirmatory work is needed to identify if dolichocephalic rabbits truly are predisposed to this specific dental abnormality. Overall, the result that brachycephaly was not associated with dental abnormalities supports the large-scale retrospective studies that also found no association between rabbit head shape and dental disease presence at multivariable level [1,2]. Perhaps the diastema in rabbits’ mouths mitigates any overcrowding issues that would otherwise lead to dental issues like those seen in brachycephalic cats and dogs [57,58,59,60].

Lower breed-estimated bodyweight was not associated with any dental abnormalities in this study, other than ocular discharge, which is of uncertain relevance, so the findings fail to support theories that dwarf rabbits are predisposed to dental disease [14,15,25,27,32,61,62]. This concurs with two larger-scale retrospective studies that also found dwarf rabbits were not predisposed to dental disease [1,3]. Behavioural reactivity to incisor and intra-oral examination also increased as breed-estimated bodyweight increased, but this likely reflected the greater difficulty of restraining large and giant rabbits.

The odds of having sharp cheek teeth edges decreased as breed-estimated bodyweight increased, indicating dwarf rabbits had higher odds of having sharp cheek teeth edges than larger rabbits. However, sharp enamel ridges on the lingual edge of mandibular cheek teeth and the buccal edge of maxillary cheek teeth are a normal finding in rabbits used to aid mastication of abrasive food [19,63], thus not considered an abnormality. Indeed, these sharp cheek teeth edges were observed in almost 75% of rabbit mouths in this study, providing further evidence of their normalcy. The clinical relevance of larger rabbits having lower odds of cheek teeth sharp edges is, once more, unclear and requires further investigation to identify if the lack of this physiological feature causes masticatory issues in larger rabbits.

### 4.2. Signalment-Related Risk Factors for Dental Abnormalities

Male rabbits had higher odds than female rabbits of having any incisor abnormality, including unhealthy, slanted, or curved incisor occlusal surfaces, and males had higher scores for behavioural reactivity to intra-oral examination than female rabbits. In contrast, female rabbits had higher odds of having long first cheek teeth than male rabbits—another finding of unclear clinical relevance. Previous studies have mostly identified that male rabbits were predisposed to dental abnormalities [1,2,3,4,64], although a few others found null associations between sex and dental abnormalities [6,7,65]. Male rabbits produce significantly greater forces and torques in their masseter muscles than females [66], perhaps explaining some differences in the presence of dental abnormalities between the sexes.

It is unclear why males appeared predisposed to slanted or curved incisor occlusal surfaces specifically, especially as that variable was another that attained Poor inter-observer agreement. The aetiology of slanted or curved incisor occlusal surfaces in rabbits is scarcely described, but it has been suggested that incisor slanting can occur if rabbits preferentially chew on only one side of the mouth, avoiding contact with contralateral painful lesions during the late stages of PSADD [8,18]; similar imbalanced incisor wear is seen in horses with unilateral cheek teeth disorders [67,68,69,70]. Alternatively, slanting or curvature could potentially occur due to repetitive biting of cage bars or consistently drinking from metal waterspouts from a particular angle. None of these factors would necessarily be expected to be more common in male rabbits than females. Future research, therefore, could assess the aetiology of slanted incisors and whether they co-occur with dental disease affecting only one side.

As rabbit age increased, the odds of having any cheek teeth abnormality, ocular discharge, and cheek teeth step or wave mouth increased. Increasing age has been reported as a dental disease risk factor in numerous studies [1,3,4,5,12], which is understandable considering the progressive nature of acquired dental disease [63].

Breed differences were not intended to be compared in this study, but fur length was included in models to help explain variance between breed types. Fur length showed significant associations with different dental disease signs in all directions and thus may have reflected various breed differences. One previous study found rex-type fur to be protective of dental disease [1], but, in the current study, rex-type fur (categorised as ‘very shorthair’) was not found protective. In fact, seven of the nine models that included fur length identified rabbits with very short or short fur as having higher odds of dental abnormalities than those with long or semi-long fur. Any association between fur length and dental disease does not seem robust, therefore requiring confirmation through further work.

### 4.3. Limitations

The clinical relevance was unclear for some of the dental abnormality variables that were mild or attained Poor inter-observer agreement. Risk factors for more obvious intermediate to end-stage dental disease could not be fully assessed in this population for several reasons. Firstly, the population of pedigree rabbits examined in this study were in good condition as they were predominantly bred specifically for exhibition, highlighted by the multivariable analyses because 33 of the 47 dental abnormality outcome variables were too rare to be tested. The median age of rabbits was just 1.29 years, so most rabbits may have been too young for dental abnormalities caused by PSADD to have developed yet, since the odds have generally been shown to increase as rabbits age [1,3,4,5,6]. Secondly, the study did not include formal diagnosis, and conscious oral examination may not always provide a sufficient view to diagnose all dental abnormalities. Elongation of sub-gingival tooth roots is usually the first indication of acquired dental disease [8,34], but this cannot be assessed via direct visual oral examination. Finally, we may have unavoidably missed some severe dental cases, because the most behaviourally reactive rabbits to oral examination (potentially due to pain) were excluded from the dataset for ethical reasons. In future studies, subject to ethical considerations, skull radiography or CT imaging could help accurately assess the full extent of dental abnormalities [16,61].

Generalising these results to companion rabbits is not advised as genetics and management practices will vary between the populations. The study population here was also non-randomly selected, so may not represent all pedigree rabbits in the UK. Breeders volunteered their rabbits, and—again for ethical reasons—were fully aware of the study aims in order to give valid consent to participate. There could, therefore, have been some bias in which rabbits were volunteered but, as dental disease is largely a hidden disease [3,5,12], breeders will not necessarily have known whether or not their rabbits were affected.

### 4.4. Implications for Improving Rabbit Dental Health

Very few conformational risk factors were found in this study, and none of those with clear clinical relevance reached statistical significance. This lack of association is unlikely to be due to insufficient statistical power, because the sample size exceeded those indicated by the sample size calculations, and because the prevalence of incisor abnormalities (28.97%; Table 5) and cheek teeth abnormalities (31.72%; Table 6) exceeded our most conservative estimate (15%; [1]). It is possible that the magnitude of any conformation effect on dental abnormalities was smaller than this study was designed to detect, but then the clinical significance of such an effect may consequently be relatively minor.

Given that the largest systematic studies of clinical populations have similarly found no statistically significant associations between ear, head, or body-size conformation and dental disease at multivariable level [1,2], it seems that these conformations may not be important risk factors for dental disease in rabbits. Therefore, there appears to be little scope for improving rabbit dental health by generically recommending breeders to avoid breeding from rabbits of these conformations, and instead direct veterinary assessment of dental health should be used to guide breeding decisions. However, even this recommendation may be difficult in practice because many breeders have large numbers of rabbits, and/or live in rural areas, without access to a veterinarian willing to see rabbits and conduct site visits. Rabbit breeders and veterinarians need to work together to develop a solution to this considerable challenge.

It is still advisable not to breed from rabbits affected by maxillary brachygnathism as this is hereditary [33,36,71], and the very low instance of incisor malocclusion in this population (0.23%; Table 5) suggests that BRC breeders may already have been effectively selecting against this abnormality. There may also exist a genetic predisposition to acquired dental disease that cannot yet be ruled out.

Rabbit exhibitions perhaps offer a further route to improve dental health in pedigree rabbits. Rabbit show judges could be trained to inspect incisors more carefully and palpate the maxillae and mandibles of rabbits to detect bone alterations from cheek teeth root overgrowth. They could also advise breeders to take potentially affected rabbits to a veterinarian, as was recommended by Korn et al. [24], or have a veterinarian attend shows to provide health checks whilst the rabbits are already out of their home environment.

These findings emphasise that, regardless of a rabbit’s conformation, proper husbandry is likely to be crucial for dental health. Feeding of good-quality long-stem hay or grass ad libitum, fresh leafy vegetables, and a small number of pellets supplemented with calcium and vitamin D is advised to prevent dental disease [3,4,53,65,72]. Muesli and high sugar treats, including bread or breakfast cereals for humans, are not recommended due to their associations with dental disease and other illnesses [72,73,74,75]. Other environmental factors, such as a lack of enrichment and lack of conspecific companionship that can cause bar-biting [76,77,78,79] and consequent dental damage, could also be a contributing factor, so breeders should ensure that they adopt appropriate husbandry practices to promote good dental health.

## 5. Conclusions

Dental abnormalities in rabbits have a complex and multifactorial aetiology. Further evidence that brachycephaly does not significantly predispose to dental abnormalities has been provided. Lop-eared and dwarf rabbits may carry higher odds of ocular discharge, and dolichocephalic rabbits may be predisposed to cheek teeth step or wave mouth, but the clinical relevance of these findings is unclear. Dental disease is often difficult to detect so breeders and veterinarians are encouraged to work together to enable all pedigree rabbits to have access to routine oral examinations. Breeders wanting to select for good dental health should avoid breeding from rabbits with maxillary brachygnathism and should provide diets and environments that protect against PSADD, but further research is needed to investigate the hereditability of other dental abnormalities. In particular, research into conformational relationships with clinically relevant ocular discharge, and into the aetiology and clinical significance of slanted incisor occlusal surfaces and step or wave mouth, could be of value, especially if skull radiography or CT imaging can be ethically used to accurately assess the full extent of dental abnormalities.

## Figures and Tables

**Table 1:** Descriptive statistics of demographic characteristics and breeds in 435 pedigree rabbits examined at British Rabbit Council shows and studs.

Variable	Categories	Number of Rabbits	Percentage of Total Rabbits Examined (%)
Sex	Male	275	63.22
	Female	158	36.32
	Missing	2	0.46
Neuter status	Entire	428	98.39
	Neutered	5	1.15
	Missing	2	0.46
Breed ^a^	Miniature Lop	71	16.32
	Netherland Dwarf	55	12.64
	Miniature Rex	41	9.43
	Dwarf Lop	38	8.74
	Angora (English)	28	6.44
	French Lop	22	5.06
	Others ^a^	180	41.37
Ear type	Erect	266	61.15
	Lop	169	38.85
Head shape	Very brachycephalic	20	4.60
	Brachycephalic	180	41.38
	Mesocephalic	90	20.69
	Slightly dolichocephalic	85	19.54
	Dolichocephalic	59	13.56
	Missing	1	0.23
Fur length	Very shorthair	53	12.18
	Shorthair	158	36.32
	Medium hair	179	41.15
	Long and semi-longhair	45	10.34

^a^ Breeds with counts of 20 or more are reported, with the remaining breeds grouped into ‘Others’.

**Table 2:** Inter-observer agreement testing for head shape and the 15 outcome variables with sufficient numbers for testing.

Variable	Percentage Agreement	Test	Agreement Coefficient	*p*-Value	Agreement Threshold
Head shape ^a^	95.00	Kendall’s W	0.970	0.008 *	Excellent
Ocular discharge	65.00	Cohen’s Kappa	0.286	0.068	Poor
Any incisor abnormality	84.21	Cohen’s Kappa	0.617	0.007 *	Substantial
Incisor malformation: any	100.00	Cohen’s Kappa	1.000	<0.001 *	Excellent
Incisor malformation: ribbing	100.00	Cohen’s Kappa	1.000	<0.001 *	Excellent
Incisor length ^b^	100.00	Cohen’s Kappa	1.000	<0.001 *	Excellent
Incisor occlusal surface: unhealthy	78.95	Cohen’s Kappa	0.377	0.084	Poor
Incisor occlusal surface: slanted or curved	78.95	Cohen’s Kappa	0.377	0.084	Poor
Any cheek teeth abnormality	77.78	Cohen’s Kappa	0.357	0.130	Poor
First cheek teeth length: short	94.44	Cohen’s Kappa	0.640	0.004 *	Substantial
First cheek teeth length: long	88.89	Cohen’s Kappa	0.437	0.063	Moderate
Cheek teeth sharp edges	94.44	Cohen’s Kappa	0.640	0.004 *	Substantial
Oral mucosa lesion: any	94.44	Cohen’s Kappa	0.640	0.004 *	Substantial
Oral mucosa lesion: laceration	100.00	Cohen’s Kappa	1.000	<0.001 *	Excellent
Reactivity to incisor examination: Any	90.00	Cohen’s Kappa	0.444	0.047 *	Moderate
Any and increasing reactivity to intra-oral examination	40.00	Kendall’s W	0.627	0.202	Substantial

* Indicates significant *p*-value; ^a^ Head shape was not a dental abnormality outcome variable but was included in inter-observer agreement testing to assess reliability of the head shape scale; ^b^ Incisor length was analysed as a binary variable due to rarity.

**Table 3:** General clinical signs of dental disease in 435 pedigree rabbits examined at British Rabbit Council shows and studs.

Variable	Number of Rabbits	Percentage of Study Population (%)
Wet fur	13	2.99
Facial asymmetry	0	0.00
Exophthalmos	0	0.00
Ocular discharge	146	33.56
Head tilt	1	0.23
Maxilla abnormality	3	0.69
Mandible abnormality	1	0.23

**Table 4:** Reactivity to examination of incisors, cheek teeth, and the oral cavity in 435 pedigree rabbits examined at British Rabbit Council shows and studs.

Examination	Behavioural Reaction	Number of Rabbits That Reacted	Percentage That Reacted (%)
Incisor	Any	95	21.84
	Move head	63	14.48
	Move body	44	10.11
	Freeze	7	1.61
	Rear	21	4.83
	Aggression	6	1.38
	Vocalisation	1	0.23
	Thumping	0	0.00
	Shaking head briefly	0	0.00
	Pawing at face	0	0.00
	Unexaminable	9	1.84
Intra-oral	Any	429	98.62
	Chewing otoscope cone minimally	338	77.70
	Chewing otoscope cone excessively	50	11.49
	Move head	116	26.67
	Move body	121	27.82
	Freeze	9	2.07
	Rear	60	13.79
	Clench teeth	24	5.52
	Aggression	20	4.60
	Vocalisation	4	0.92
	Thumping	0	0.00
	Pawing at face	0	0.00
	Shaking head briefly	2	0.46
	Unexaminable	55	12.64

**Table 5:** Incisor abnormalities identified in 435 pedigree rabbits examined at British Rabbit Council shows and studs. These data were missing for 12 rabbits.

Variable	Descriptor	Number of Rabbits Affected	Percentage of Study Population (%)
Able to examine all incisors	Yes	423	97.24
Incisor abnormality	Any	126	28.97
Incisor malformation	Any	10	2.30
	Missing teeth	0	0.00
	Fracture	3	0.69
	Ribbing	7	1.61
Incisor length	Short	0	0.00
	Long	7	1.61
Incisor colour	Abnormal	8	1.84
Incisor occlusal surface	Any unhealthy occlusal surface	111	25.52
	Rough	3	0.69
	Slanted or curved	111	25.52
Incisor malocclusion	Any	5	1.15
	Misdirection	4	0.92
	Maxillary brachygnathism	0	0.00
	Mandibular brachygnathism	0	0.00
	Incisors touching	1	0.23

**Table 6:** Cheek teeth abnormalities identified in 435 pedigree rabbits examined at British Rabbit Council shows and studs. Number of ‘missing’ in variables is not constant due to rabbits without all cheek teeth present and three records where a variable was not recorded by an observer.

Variable	Descriptor	Number of Rabbits Affected	Percentage of Study Population (%)
Able to examine all cheek teeth	Yes	379	87.13
Any cheek teeth abnormality ^a^	Yes	138	31.72
	Missing data	56	12.87
Cheek teeth malformation: any	Yes	5	1.15
	Missing data	56	12.87
Cheek teeth malformation: missing teeth	Yes	3	0.69
	Missing data	56	12.87
Cheek teeth malformation: fracture	Yes	4	0.92
	Missing data	56	12.87
Cheek teeth colour	Abnormal	12	2.76
	Missing data	59	13.56
First cheek teeth length	Short	72	16.55
	Long	70	16.09
	Missing data	47	10.80
Caudal cheek teeth length	Short	27	6.21
	Long	17	3.91
	Missing data	57	13.10
Cheek teeth spurs	Yes	15	3.45
	Missing data	58	13.33
Cheek teeth step or wave mouth	Yes	54	12.41
	Missing data	60	13.79
Cheek teeth sharp edges ^a^	Yes	323	74.25
	Missing data	59	13.56

^a^ Sharp edges were not classed as an abnormality.

**Table 7:** Clinical signs of dental disease affecting the oral mucosa and tongue in 435 pedigree rabbits examined at British Rabbit Council shows and studs. These data were missing for 54 rabbits.

Variable	Number of Rabbits	Percentage of Study Population (%)
Able to examine oral cavity ^a^	381	87.59
Oral lesion: any	25	5.75
Hyperaemia	16	3.68
Bleeding	8	1.84
Laceration	3	0.69
Ulceration	1	0.23
Purulent discharge	0	0.00
Hyperplasia	2	0.46

^a^ Two rabbits tolerated examination of the oral cavity but not of all cheek teeth.

**Table 8:** Statistically significant conformational and signalment-related results and trends of binary logistic and linear generalised estimating equation models for dental abnormality outcome variables in 435 pedigree rabbits examined at British Rabbit Council shows and studs.

Predictor	Effect Direction	Outcome	Odds Ratio or B Coefficient	95% CI	*p*-Value *
Ear type	Lop-eared rabbits had higher odds than erect-eared rabbits	Ocular discharge	4.034	1.475–11.030	0.007
		Unhealthy incisor occlusal surface	1.889	1.088–3.279	0.024 ^FDR^
		Slanted or curved incisor occlusal surface	1.862	1.079–3.214	0.026 ^FDR^
	Erect-eared rabbits had higher odds than lop-eared rabbits	Any reactivity to incisor examination	1.666	1.118–2.338	0.003
Head shape	As head shape lengthened, the odds increased	Cheek teeth step or wave mouth	1.394	1.114–1.744	0.004
		Caudal cheek teeth length: short	1.450	1.017–2.068	0.040 ^FDR^
	As head shape lengthened, the odds decreased	No outcome variables reached statistical significance	n/a	n/a	n/a
Breed-estimated bodyweight	As breed-estimated bodyweight increased, the odds/scores increased	Ocular discharge	1.962	1.466–2.625	<0.001
		Any reactivity to incisor examination	1.394	1.171–1.660	<0.001
		Increasing reactivity to incisor examination ^a^	0.361	0.191–0.532	<0.001
		Any and increasing reactivity to intra-oral examination ^a^	0.385	0.145–0.624	0.002
	As breed-estimated bodyweight increased, the odds decreased	Any cheek teeth abnormality	0.871	0.766–0.991	0.036 ^FDR^
		Cheek teeth sharp edges	0.632	0.518–0.771	<0.001
Sex	Males had higher odds/scores than females	Any incisor abnormality	2.057	1.170–3.618	0.012
		Unhealthy incisor occlusal surface	2.284	1.314–3.969	0.003
		Slanted or curved incisor occlusal surface	2.229	1.260–3.941	0.006
		Any reactivity to incisor examination	1.757	1.032–2.990	0.038 ^FDR^
		Any and increasing reactivity to intra-oral examination ^a^	0.468	0.165–0.771	0.003
	Females had higher odds than males	First cheek teeth length: long	2.175	1.324–3.573	0.002
		Caudal cheek teeth length: short	2.186	1.013–4.718	0.046 ^FDR^
Age	As age increased, the odds increased	Ocular discharge	1.203	1.055–1.371	0.006
		Any cheek teeth abnormality	1.483	1.214–1.810	<0.001
		Cheek teeth step or wave mouth	1.530	1.311–1.785	<0.001
	As age increased, the scores decreased	Increasing reactivity to incisor examination	−0.130 ^a^	−0.243–−0.017	0.024 ^FDR^
Fur length	Very shorthair rabbits had higher odds than rabbits with other fur lengths	Any cheek teeth abnormality	Table S4 ^b^	Table S4 ^b^	<0.001 *
		Cheek teeth step or wave mouth	Table S4 ^b^	Table S4 ^b^	<0.001 *
	Shorthair rabbits had higher odds than rabbits with other fur lengths	No outcome variables reached statistical significance	n/a	n/a	n/a
	Medium hair rabbits had higher odds than rabbits with other fur lengths	Ocular discharge	Table S4 ^b^	Table S4 ^b^	0.049 ^FDR^
	Long and semi-longhair rabbits had higher odds/scores than rabbits with other fur lengths	Any incisor abnormality	Table S4 ^b^	Table S4 ^b^	<0.001 *
		Incisor occlusal surface: Unhealthy	Table S4 ^b^	Table S4 ^b^	0.001 *
		Incisor occlusal surface: Slanted or curved	Table S4 ^b^	Table S4 ^b^	0.002 *
		First cheek teeth length: Short	Table S4 ^b^	Table S4 ^b^	0.001 *
		First cheek teeth length: Long	Table S4 ^b^	Table S4 ^b^	<0.001 *
		Caudal cheek teeth length: Short	Table S4 ^b^	Table S4 ^b^	<0.001 *
		Increasing reactivity to incisor examination ^a^	Tables S4 and S5 ^b^	Tables S4 and S5 ^b^	<0.001 *

Abbreviation: CI, confidence interval; n/a, not applicable; * *p* < 0.019—the significance threshold following controlling for the false discovery rate; ^FDR^ indicates nonsignificant trends reported for completeness, after controlling for the false discovery rate; ^a^ indicates a Beta coefficient rather than odds ratio as this variable was recorded as a scale and tested with a linear generalised estimating equation; ^b^ Significant results for detailed fur-length pairwise comparisons, methodological factors, and all non-significant results can be seen in Supplementary Materials, Tables S4 and S5.

## Data Availability

The original data presented in the study are openly available in FigShare at https://doi.org/10.6084/m9.figshare.28368476. Breed names have been removed, and breed-estimated bodyweight for each rabbit has been rounded to the nearest whole number in this dataset to preserve anonymity of breeders.

## Supplementary Materials

The following supporting information can be downloaded at: https://www.mdpi.com/article/10.3390/ani15070980/s1, Table S1: Reactivity ethogram for behaviours observed during, or within 30 s of oral examination of 435 rabbits at British Rabbit Council shows and studs; Table S2: Descriptive criteria for dental abnormalities. Definitions created with adaptions from Capello (2016) [13], Johnson and Burn (2019) [7], Studdert et al. (2021) [80], and Jackson et al. (in press) [11]; Table S3: Reproduction of the assessment checklist used to record signalment, general clinical information, ear abnormalities, dental abnormalities, and free-text comments during examination of 435 British Rabbit Council pedigree rabbits. Hyperaemia of the tongue and oral mucosa was subsequently renamed as ‘erythema’; Table S4: Results of binary logistic generalised estimating equation models for ocular discharge and dental abnormalities in 435 pedigree rabbits examined at British Rabbit Council shows and studs. [Forced] indicates this variable was not significant at univariable level but was included in the final multivariable model as a variable of a priori interest; Table S5: Results of linear generalised estimating equation models for reactivity during incisor and intra-oral examination in 435 pedigree rabbits examined at British Rabbit Council shows and studs.
