# Supplementary material for: Rabbit Dental Abnormalities: Investigation of Conformational Risk Factors in a Pedigree Rabbit Population
Source: Animals (Basel). 2025 Mar 28;15(7):980. doi: 10.3390/ani15070980 (PMC11987845; doi:10.3390/ani15070980)
Supplement: Supplementary file 1 [file animals-15-00980-s001.zip › animals-3491833-supplementary.pdf]

Supporting Information

Rabbit Dental Abnormalities: Investigation of Conformational Risk Factors in a Pedigree Rabbit Population

Table S1. Reactivity ethogram for behaviours observed during, or within 30 seconds of oral examination of 435 rabbits at British Rabbit Council shows and studs.

Reaction	Definition of behaviour
Freezing	The rabbit is motionless for at least 10 seconds. They may press their ears toward their body (if erect-eared), and their head and body may be pressed towards the table. If manipulated, the rabbit's posture remains relatively stiff.
Aggression	The rabbit displayed boxing behaviour and/or bites, or displays an attempt to bite (indicated by, for example, lunging forwards with an open mouth), the examiner or restrainer during any part of the physical examination, or the otoscope cone before entry into the mouth or after removal from the mouth during oral examination.
Vocalising	Emitting an audible sound during restraint and/or otoscopic examination.
Thumping	Rapid and sudden stamping of one or both hindfeet against the table surface, at least once.
Shaking head	Shaking of the head from side-to-side. Persists for less than five seconds, or occurs on three or fewer separate occasions.
Moving head	The rabbit moves their head away from the otoscope, examiner, or restrainer, by turning their head sideways and/or pulling head backward into the neck and/or body, without moving whole body. This does not include head movement caused by manipulation from the examiner or restrainer.
Moving body	The rabbit moves their body away from the otoscope, examiner, or restrainer by reversing backwards, and/or or turning body around, and/or attempting to run away from observer, with or without paws remaining on the table surface. This does not include body movement caused by manipulation from the examiner or restrainer.
Rearing up	Rabbit suddenly lifts the cranial part of their body upwards and away from the otoscope and observer. The rabbit may twist their body or arch their back.
Pawing at mouth	Rubbing of one or both forelimbs against either side of the mouth, unrelated to the normal grooming sequence.
Clench teeth	Holding the mouth closed upon, or after, insertion of the otoscope that prevents full, or any, visualisation of the dental arcades. The rabbit may clench their teeth onto the otoscope cone to prevent its movement.
Chewing otoscope cone minimally	Chewing the otoscope cone upon insertion into the oral cavity for less than five seconds, that does not prevent full otoscopic examination.
Chewing otoscope cone excessively	Chewing the otoscope cone upon insertion into the oral cavity for over five seconds, or continuously throughout oral examination, but that does not prevent full otoscopic examination.
Un-examinable	A reaction to intra-oral examination that prevents the observer from looking inside the mouth using the otoscope, that reaches the ethical threshold for

	examination. For example, abnormally excessive or continuous chewing, clamped mouth, or moving away from examiner repeatedly.
Other	Any reaction not described in the ethogram, accompanied with a description.
None	The rabbit displays none of the listed behaviour but is not freezing.

Table S2. Descriptive criteria for dental abnormalities. Definitions created with adaptions from Capello (2016), Johnson and Burn (2019), Studdert *et al.* (2021), and Jackson *et al.* (in press).

Category	Abnormality	Descriptor	Definition
General signs of illness	Wet fur	n/a	Wet fur at time of examination, or crusty and matted fur indicating recent wetness, anywhere on the rabbit's body and face.
	Head tilt	n/a	When looking from the front at the same height as the rabbit, the rabbit's head appears tilted to one side, with one eye at a lower height than the other.
	Facial asymmetry	n/a	When looking from the front at the same height as the rabbit, the rabbits' face appears lopsided, uneven, or not symmetrical. May include facial paralysis or muscle contracture.
	Ocular discharge	n/a	Presence of any amount of discharge of any type from the eye, or a crusting or damp area below the eye at time of examination. May include hyperlacrimation, epiphora, conjunctivitis, and/or dacryocystitis, from any cause including foreign bodies.
	Exophthalmos	n/a	Bulging eyeball that protrudes from the usual position within the socket.
	Maxilla abnormality	n/a	Any swellings or changes to the usual smooth feeling of the maxilla on gentle palpation.
	Mandible abnormality	n/a	Any swellings or changes to the usual smooth feeling of the mandible on gentle palpation.
Incisor abnormalities	Malformation	Any	A malformation to at least one incisor itself. An encompassing term for missing teeth, fractures, and/or ribbing in this study.
		Missing teeth	At least one completely absent incisor, with no part of the tooth visible on gross visual examination.
		Fracture	At least one fractured incisor. May be completely broken or shattered, fractured at any angle including longitudinally, or have a part visibly chipped or cracked off, leaving an abnormally shaped tooth.
		Ribbing	Horizontal enamel ridges across at least one incisor. May be subtle or deep grooves.
	Length	Short	At least one incisor is abnormally short compared to the length of typical rabbit incisors.
		Normal	The incisors are of a normal, expected length.
		Long	At least one incisor is abnormally long compared to the length of typical rabbit incisors.
	Colour	0	The incisors are a healthy, opaque, off-white colour.

		1	At least one incisor appears more yellow or more translucent than a normal, expected colour.
		2	At least one incisor appears to be a dark yellow to brown, or black colour.
		Pink	At least one incisor appears to be pale pink in colour (possibly indicative of pulp exposure).
	Occlusal surface	Unhealthy	At least one incisor does not have a smooth, horizontal occlusal surface. An encompassing term for a rough, slanted, and/or curved occlusal surface in this study.
		Rough	When looking from the front, the occlusal surface of at least one incisor is rough or bumpy, not smooth and horizontal like that of a normal incisor. The affected incisor may or may not have lost the chisel-like shape visible on the lateral aspect. This may be from any cause, including fractured incisors.
		Slanted or curved	When looking from the front, the occlusal surface of at least one incisor is slanted to one side or has a concave or convex curve in the centre, not smooth and horizontal like that of a normal incisor. The affected incisor may or may not have lost the chisel-like shape visible on the lateral aspect.
	Malocclusion	Any	Incisors that grow in any abnormal position, resulting in imperfect positioning of teeth when the mouth is closed. An encompassing term for misdirection, incisors touching, maxillary brachygnathia, and/or mandibular brachygnathia in this study.
		Misdirection	One or more incisors growing at any abnormal angle or any inappropriate direction from the apical root of the tooth. This may be from a congenital jaw defect or a previous traumatic injury.
		Incisors touching	The incisor occlusal surfaces are touching each other instead of the maxillary incisors sitting just rostral to (in front of) mandibular incisors. Can be an early sign of maxillary brachygnathism, but a young rabbit may grow out of this.
		Maxillary brachygnathism	An underbite. The lower jaw length is longer than the upper jaw resulting in mandibular incisors protruding beyond the maxillary incisors (also known as mandibular prognathism in other species).
		Maxillary prognathism	An overbite. The upper jaw length is substantially longer than the lower jaw resulting in maxillary incisor overgrowth and usually curling of the mandibular incisors as they reach the hard palate of the mouth (also known as maxillary prognathism in other species).
Cheek teeth abnormalities	Malformation	Any	A malformation to at least one cheek tooth itself. An encompassing term for missing teeth and/or fractures in this study.

		Missing teeth	At least one completely absent cheek tooth, with no part of the tooth visible on gross visual examination.
		Fracture	At least one fractured cheek tooth. May be completely broken or shattered, fractured at any angle including longitudinally, or have a part visibly chipped or cracked off, leaving an abnormally shaped tooth.
	Colour	0	The cheek teeth are generally a healthy, opaque, off-white colour. Some yellow or brown staining is normal, particularly in the longitudinal enamel ridges.
		1	At least one cheek tooth appears more yellow or darker than a normal, expected colour.
		2	At least one cheek tooth appears to be a dark yellow to brown, or black colour.
	Length (first cheek teeth)	Short	When looking from the front at the same height as the rabbit, the first cheek tooth is abnormally short. The occlusal surface sits lower than the caudal cheek teeth in that quadrant if the caudal cheek teeth are of normal length.
		Normal	When looking from the front at the same height as the rabbit, the first cheek tooth is of a normal, expected length. The occlusal surface is at the same height as the caudal cheek teeth in that quadrant if the caudal cheek teeth are of normal length.
		Long	When looking from the front at the same height as the rabbit, the first cheek tooth is abnormally long. The occlusal surface sits higher than the caudal cheek teeth in that quadrant if the caudal cheek teeth are of normal length.
	Length (caudal cheek teeth)	Short	When looking from the front at the same height as the rabbit, excluding the first cheek tooth, all caudal cheek teeth are abnormally short. Occlusal surfaces sit lower than would be expected in a normal rabbit.
		Normal	When looking from the front at the same height as the rabbit, excluding the first cheek tooth, all caudal cheek teeth are of a normal, expected length. Occlusal surfaces sit at the expected height for a normal rabbit.
		Long	When looking from the front at the same height as the rabbit, excluding the first cheek tooth, all caudal cheek teeth are abnormally long. Occlusal surfaces sit higher than would be expected in a normal rabbit.
	Spurs	n/a	An abnormal, overgrown protrusion from the edge of at least one cheek tooth, typically protruding towards the tongue (lingually) for mandibular cheek teeth and protruding towards the cheek (buccally) for maxillary cheek teeth.

	Step or wave mouth	n/a	The occlusal surfaces of cheek teeth are of differing heights, and/or, when viewed from the front, cheek teeth do not all appear in line with one or more laterally deviated from a normal position.
	Sharp edges	n/a	A normal, sharp vertically directed enamel point of at least one cheek tooth at buccal and/or lingual edges.
Oral lesions	Tongue	Any	A lesion of any kind at any location on the tongue itself. An encompassing term for hyperaemia, bleeding, laceration, ulceration, purulent discharge, and/or hyperplasia in this study.
		Erythema (initially called ‘hyperaemia’ on the assessment checklist)	Reddening or erythema in patches of any size on the tongue.
		Bleeding	Any active external escape of blood on the tongue.
		Laceration	A wound or tear in the tongue, of any size. Does not have to be actively bleeding.
		Ulceration	An inflamed damaged area, defect, or break in the epithelial surface of the tongue. May or may not be infected or have a covering of inflammatory exudate.
		Purulent discharge	An area (or areas) or wound on the tongue producing exudate that appears opaque and thick with a white to yellow colour.
		Hyperplasia	An area (or areas) of abnormally increased volume of tongue tissue.
	Other oral mucosa	None	A lesion of any kind at any location on the oral mucosa, other than the tongue. An encompassing term for hyperaemia, bleeding, laceration, ulceration, purulent discharge, and/or hyperplasia in this study.
		Erythema (initially called ‘hyperaemia’ on the assessment checklist)	Reddening or erythema in patches of any size on any part of the oral mucosa, other than the tongue.
		Bleeding	Any active external escape of blood on any part of the oral mucosa, other than the tongue.
		Laceration	A wound or tear in any part of the oral mucosa other than the tongue, of any size. Does not have to be actively bleeding.

		Ulceration	An inflamed damaged area, defect, or break in the epithelial surface of the oral mucosa. May or may not be infected or have a covering of inflammatory exudate.
		Purulent discharge	An area or wound on the oral mucosa producing exudate that appears opaque and thick with a white to yellow colour.
		Hyperplasia	An area (or areas) of abnormally increased volume of oral mucosal tissue, including gingival hyperplasia.

Table S2 References (numbers in square brackets correspond to references in the main text)

[7] Johnson, J.C.; Burn, C.C. Lop-eared Rabbits Have More Aural and Dental Problems than Erect-eared Rabbits: A Rescue Population Study. *Veterinary Record* **2019**, *185*, 758–758, doi:10.1136/vr.105163.

[11] Jackson, M.A.; O'Neill, D.G.; Hedley, J.; Brodbelt, D.C.; Burn, C.C. Dental Disease in Rabbits under UK Primary Veterinary Care: Clinical Management and Associated Welfare Impacts. *Veterinary Record* **2025**, [*in press*].

[13] Capello, V. Diagnostic Imaging of Dental Disease in Pet Rabbits and Rodents. *Veterinary Clinics of North America: Exotic Animal Practice* **2016**, *19*, 757–782, doi:10.1016/j.cvex.2016.05.001.

[80] Studdert, V.P.; Gay, C.C.; Hinchcliff, K.W. *Saunders Comprehensive Veterinary Dictionary*, 5th ed.; Elsevier: St. Louis, USA, 2021; ISBN 978-0-7020-7463-9.

Table S3. Reproduction of the assessment checklist used to record signalment, general clinical information, ear abnormalities, dental abnormalities, and free-text comments during examination of 435 British Rabbit Council pedigree rabbits. Hyperaemia of the tongue and oral mucosa was subsequently renamed as 'erythema'.

**Date:**	**Show/stud name:**	**Sex/Neuter status:**
**Examination number:**	**Breed:**	**Year of birth:**
**Examiner's initials:**	**Ring number:**	**Age bracket:**

**Head shape (1-5)**		**Ocular discharge**	Left- Y□ N□	Right- Y□ N□
**Wet fur**	Y□ N□ Location:	**Exophthalmos**	Left- Y□ N□	Right- Y□ N□
**Head tilt**	Y□ N□ Lower side:	**Maxilla abnormal**	Left- Y□ N□	Right- Y□ N□
**Facial asymmetry**	Y□ N□ Comment:	**Mandible abnormal**	Left- Y□ N□	Right- Y□ N□

	**Left**		**Right**	
**Ear base swelling (✓/x)**				
**Pinna- dermatological change**	None Scaling out Scaling in Alopecia Erythema Exudate Crust Ulcer Nodule		None Scaling out Scaling in Alopecia Erythema Exudate Crust Ulcer Nodule	
**Pinna- trauma**	None Abrasion Exudate Healed laceration Fresh laceration		None Abrasion Exudate Healed laceration Fresh laceration	
**Diverticulum discharge (✓/x, col)**				
**Stenosis (0-3)**				
**Ear canal colour**	None Reddening Erythema		None Reddening Erythema	
**Discharge- flaky, crumbly, dry (0-4)**	Upper-	Lower-	Upper-	Lower-
**Discharge- lining, sticky, wet (0-4)**	Upper-	Lower-	Upper-	Lower-
**Discharge- pink, red, scabs (0-4)**	Upper-	Lower-	Upper-	Lower-
**Reactivity to ear exam**				
**Comments**				
**Able to examine?**	Y□ N□		Y□ N□	

Incisors		**Upper**	**Lower**	All normal? □
	**Malformations**	None Missing teeth Fracture Ribbing	None Missing teeth Fracture Ribbing	
	**Length (S/N/L)**			
	**Colour (0-2 or P)**			
	**Occlusal surface**	Healthy Rough Slanted or curved	Healthy Rough Slanted or curved	
	**Malocclusion**	None Misdirection Underbite Overbite Incisors touching		
	**Comments (✓/x)**			
	**Able to examine?**	Y□ N□	Y□ N□	

CT- first PM		**Left**		**Right**		All normal? □
	**Malformations**	None Missing teeth Fracture		None Missing teeth Fracture		
	**Colour (0-2)**					
	**Length (S/N/L)**	Upper-	Lower-	Upper-	Lower-	
	**Spurs (✓/x)**	Upper-	Lower-	Upper-	Lower-	
	**Sharp edges (✓/x)**	Upper-	Lower-	Upper-	Lower-	
	**Comments (✓/x)**					
	**Able to examine?**	Y□ N□		Y□ N□		

CT- all others		**Left**		**Right**		All normal? □
	**Malformations**	None Missing teeth Fracture		None Missing teeth Fracture		
	**Colour (0-2)**					
	**Length (S/N/L)**	Upper-	Lower-	Upper-	Lower-	
	**Step/wave mouth (✓/x)**	Upper-	Lower-	Upper-	Lower-	
	**Spurs (✓/x)**	Upper-	Lower-	Upper-	Lower-	
	**Sharp edges (✓/x)**	Upper-	Lower-	Upper-	Lower-	
	**Comments (✓/x)**					
	**Able to examine?**	Y□ N□		Y□ N□		

Mouth		
	**Tongue lesion**	None Hyperaemia Bleeding Laceration Ulceration Purulent discharge Hyperplasia
	**Other oral lesion**	None Hyperaemia Bleeding Laceration Ulceration Purulent discharge Hyperplasia
	**Able to examine?**	Y□ N□

**Reactivity to incisor exam**		
**Reactivity to CT exam**	*Left-*	*Right-*
**Comments (✓/x)**		

Any other comments:

Record uploaded □

Table S4. Results of binary logistic generalised estimating equation models for ocular discharge and dental abnormalities in 435 pedigree rabbits examined at British Rabbit Council shows and studs. [Forced] indicates this variable was not significant at univariable level but was included in the final multivariable model as a variable of *a priori* interest.

Outcome variable	Predictor variable	Reference category	Comparison category	Variable Wald Chi-square	Odds ratio	95% confidence interval	p-value
Ocular discharge (n=430)	Examiner	MJ	MB	36.632	3.950	2.532 – 6.164	<0.001*
	Sex	Male	Female	1.349	0.768	0.492 – 1.199	0.245
	Ear type	Erect	Lop	7.383	4.034	1.475 – 11.030	0.007*
	Fur length	Overall model		7.849			0.049^{FDR}
		Medium hair	Very shorthair	5.404	4.708	1.275 – 17.381	0.020^{FDR}
			Shorthair	1.278	1.619	0.702 – 3.731	0.258
			Long and semi-long	4.673	5.461	1.172 – 25.449	0.031^{FDR}
	Head shape	n/a	n/a	3.059	0.774	0.580 – 1.031	0.080
	Examination number	n/a	n/a	7.036	1.003	1.001 – 1.006	0.008*
	Breed-estimated bodyweight (kg)	n/a	n/a	20.551	1.962	1.466 – 2.625	<0.001*
	Age (years)	n/a	n/a	7.646	1.203	1.055 – 1.371	0.006*
Any incisor abnormality (n=418)	Sex	Female	Male	6.272	2.057	1.170 – 3.618	0.012*
	Ear type	Erect	Lop	1.963	1.498	0.851 – 2.635	0.161
	Fur length	Overall model		39.387			<0.001*
		Long and semi-long	Very shorthair	6.485	1.786	1.143 – 2.791	0.011*
			Shorthair	16.385	2.044	1.446 – 2.889	<0.001*
			Medium hair	5.455	1.921	1.111 – 3.324	0.020*
	Head shape	n/a	n/a	0.156	1.055	0.809 – 1.375	0.693
	Examination number	n/a	n/a	6.510	1.003	1.001 – 1.005	0.011*
	Breed-estimated bodyweight (kg)	n/a	n/a	0.979	0.927	0.797 – 1.078	0.323
	Age (years)	n/a	n/a	2.541	1.109	0.977 – 1.260	0.111
	Sex	Female	Male	8.580	2.284	1.314 – 3.969	0.003*

Incisor occlusal surface: Unhealthy (n=418)	Ear type	Erect	Lop	5.114	1.889	1.088 – 3.279	0.024[FDR]
	Fur length	Overall model<br>Long and semi-long	 Very shorthair<br>Shorthair<br>Medium hair	15.877<br>2.928<br>12.981<br>4.526	 1.623<br>2.031<br>1.872	 0.932 – 2.826<br>1.381 – 2.987<br>1.051 – 3.336	0.001*<br>0.087<br><0.001*<br>0.033[FDR]
	Head shape	n/a	n/a	0.054	1.029	0.808 – 1.312	0.816
	Examination number	n/a	n/a	6.794	1.003	1.001 – 1.005	0.009*
	Breed-estimated bodyweight (kg)	n/a	n/a	0.412	0.951	0.814 – 1.110	0.521
	Age (years)	n/a	n/a	1.393	1.088	0.946 – 1.252	0.238
Incisor occlusal surface: Slanted or curved (n=418)	Sex	Female	Male	7.592	2.229	1.260 – 3.941	0.006*
	Ear type	Erect	Lop	4.989	1.862	1.079 – 3.214	0.026[FDR]
	Fur length	Overall model<br>Long and semi-long	 Very shorthair<br>Shorthair<br>Medium hair	15.249<br>0.835<br>13.625<br>4.848	 1.345<br>2.058<br>1.940	 0.712 – 2.541<br>1.403 – 3.019<br>1.076 – 3.499	0.002*<br>0.361<br><0.001*<br>0.028[FDR]
	Head shape	n/a	n/a	0.169	1.054	0.820 – 1.355	0.681
	Examination number	n/a	n/a	6.907	1.003	1.001 – 1.005	0.009*
	Breed-estimated bodyweight (kg)	n/a	n/a	0.555	0.941	0.803 – 1.104	0.456
	Age (years)	n/a	n/a	1.583	1.105	0.946 – 1.290	0.208
Any cheek teeth abnormality (n=375)	Sex [forced]	Male	Female	1.922	1.338	0.886 – 2.019	0.166
	Ear type [forced]	Erect	Lop	3.168	0.563	0.299 – 1.060	0.075
	Fur length	Overall model<br>Very shorthair	 Shorthair<br>Medium hair<br>Long and semi-long	44.331<br>14.585<br>24.309<br>1.531	 3.823<br>7.708<br>1.345	 1.921 – 7.610<br>3.422 – 17.357<br>0.841 – 2.152	<0.001*<br><0.001*<br><0.001*<br>0.216
	Head shape	n/a	n/a	0.005	0.992	0.793 – 1.240	0.942
	Breed-estimated bodyweight (kg)	n/a	n/a	4.389	0.871	0.766 – 0.991	0.036[FDR]

	Age (years)	n/a	n/a	14.907	1.483	1.214 – 1.810	<0.001*
Cheek teeth sharp edges (n=372)	Examiner	MB	MJ	5.024	2.456	1.119 – 5.390	0.025[FDR]
	Sex [forced]	Male	Female	1.907	1.702	0.800 – 3.622	0.167
	Ear type [forced]	Erect	Lop	0.459	1.374	0.548 – 3.446	0.498
	Fur length[a]	Overall model	n/a	5.675	n/a	n/a	0.129
	Head shape	n/a	n/a	0.117	1.062	0.753 – 1.496	0.732
	Examination number	n/a	n/a	1.820	1.002	0.999 – 1.006	0.177
	Breed-estimated bodyweight (kg)	n/a	n/a	20.403	0.632	0.518 – 0.771	<0.001*
	Age (years) [forced]	n/a	n/a	0.017	0.982	0.742 – 1.300	0.898
Cheek teeth step or wave mouth (n=371)	Sex	Male	Female	0.628	0.812	0.486 – 1.358	0.428
	Ear type	Erect	Lop	0.105	0.851	0.322 – 2.252	0.745
	Fur length	Overall model<br>Very shorthair	 Shorthair<br>Medium hair<br>Long and semi-long	19.632<br>18.157<br>12.038<br>0.922	 4.371<br>8.271<br>1.673	 2.218 – 8.614<br>2.507 – 27.284<br>0.585 – 4.785	<0.001*<br><0.001*<br><0.001*<br>0.337
	Head shape [forced]	n/a	n/a	8.414	1.394	1.114 – 1.744	0.004*
	Age (years)	n/a	n/a	29.074	1.530	1.311 – 1.785	<0.001*
First cheek teeth length: Short (n=383)	Sex [forced]	Male	Female	2.235	1.659	0.854 – 3.223	0.135
	Ear type [forced]	Erect	Lop	0.052	0.920	0.452 – 1.874	0.819
	Fur length	Overall model<br>Long and semi-long	 Very shorthair<br>Shorthair<br>Medium hair	15.902<br>13.095<br>11.246<br>14.792	 6.025<br>4.932<br>8.068	 2.278 – 15.938<br>1.941 – 12.533<br>2.784 – 23.382	0.001*<br><0.001*<br><0.001*<br><0.001*
	Head shape [forced]	n/a	n/a	0.082	0.969	0.779 – 1.204	0.774
	Examination number	n/a	n/a	6.433	1.004	1.001 – 1.007	0.011*
	Age (years) [forced]	n/a	n/a	0.728	0.947	0.835 – 1.074	0.393
First cheek teeth length: Long (n=384)	Examiner	MB	MJ	6.132	1.848	1.137 – 3.006	0.013*
	Sex	Male	Female	9.418	2.175	1.324 – 3.573	0.002*
	Ear type [forced]	Erect	Lop	0.132	0.885	0.458 – 1.709	0.716

	Fur length	Overall model<br>Long and semi-long	 Very shorthair<br>Shorthair<br>Medium hair	60.863<br>29.709<br>59.875<br>22.129	 8.249<br>13.492<br>10.714	 3.862 – 17.616<br>6.980 – 26.080<br>3.989 – 28.779	<0.001*<br><0.001*<br><0.001*<br><0.001*
	Head shape [forced]	n/a	n/a	0.007	0.987	0.714 – 1.364	0.935
	Examination number	n/a	n/a	4.353	1.002	1.000 – 1.005	0.037[FDR]
	Breed-estimated bodyweight (kg)	n/a	n/a	1.373	0.864	0.676 – 1.104	0.241
	Age (years) [forced]	n/a	n/a	1.005	0.906	0.748 – 1.098	0.316
Caudal cheek teeth length: Short (n=374)	Sex	Male	Female	3.969	2.186	1.013 – 4.718	0.046[FDR]
	Ear type [forced]	Erect	Lop	0.462	0.696	0.245 – 1.977	0.497
	Fur length	Overall model<br>Long and semi-long	 Very shorthair<br>Shorthair<br>Medium hair	17.101<br>9.354<br>3.534<br>12.802	 2.775<br>2.343<br>5.885	 1.443 – 5.338<br>0.964 – 5.693<br>2.229 – 15.539	<0.001*<br>0.002*<br>0.060<br><0.001*
	Head shape [forced]	n/a	n/a	4.209	1.450	1.017 – 2.068	0.040[FDR]
	Examination number	n/a	n/a	3.478	1.004	1.000 – 1.009	0.062
	Age (years)	n/a	n/a	1.277	1.146	0.905 – 1.450	0.258
Oral lesion: Any (n=377)	Examiner	MB	MJ	15.974	9.096	3.077 – 26.769	<0.001*
	Sex [forced]	Male	Female	0.878	1.924	0.489 – 7.567	0.349
	Ear type	Erect	Lop	0.070	0.740	0.079 – 6.911	0.792
	Fur length[a]	Overall model	n/a	1.149	n/a	n/a	0.765
	Head shape [forced]	n/a	n/a	0.319	0.832	0.439 – 1.577	0.572
	Breed-estimated bodyweight (kg)	n/a	n/a	1.223	0.757	0.462 – 1.240	0.269
	Age (years)	n/a	n/a	0.301	1.071	0.837 – 1.371	0.583
	Examiner	MB	MJ	14.119	2.929	1.672 – 5.132	<0.001*
	Sex	Female	Male	4.311	1.757	1.032 – 2.990	0.038[FDR]

Any reactivity to incisor examination (n=430)	Examination location (show or stud)	Show	Stud	6.698	2.528	1.252 – 5.103	0.010*
	Ear type	Lop	Erect	8.740	1.666	1.118 – 2.338	0.003*
	Head shape	n/a	n/a	2.530	1.212	0.956 – 1.535	0.112
	Examination number	n/a	n/a	4.513	0.997	0.995 – 1.000	0.034[FDR]
	Breed-estimated bodyweight (kg)	n/a	n/a	13.915	1.394	1.171 – 1.660	<0.001*
	Age (years)	n/a	n/a	0.313	0.943	0.769 – 1.157	0.576

* Indicates significant p-value at the false discovery rate corrected threshold of p<0.019.

[FDR] indicates nonsignificant trends reported for completeness, after controlling for the false discovery rate.

[a] This variable was not significant at the overall model level, so the post-hoc pairwise comparisons are not reported.

Table S5. Results of linear generalised estimating equation models for reactivity during incisor and intra-oral examination in 435 pedigree rabbits examined at British Rabbit Council shows and studs.

	Predictor variable	Reference category	Comparison category	Standard error	Wald Chi-square	B coefficient	95% Wald confidence interval	p-value
Increasing reactivity to incisor examination (n=95)	Examiner	MJ	MB	0.2352	0.027	0.039	-0.422 – 0.500	0.869
	Sex	Male	Female	0.1331	0.028	-0.022	-0.283 – 0.239	0.867
	Examination location (show or stud)	Show	Stud	0.2402	17.165	0.995	0.524 – 1.466	<0.001*
	Ear type	Erect	Lop	0.3171	1.149	0.340	-0.282 – 0.961	0.284
	Fur length	Overall model			45.117			<0.001*
		Long and semi-longhair	Very shorthair	0.2222	24.846	1.107	0.672 – 1.543	<0.001*
			Shorthair	0.1126	23.254	0.543	0.322 – 0.764	<0.001*
			Medium hair	0.2909	6.450	0.739	0.169 – 1.309	0.001*

	Head shape	n/a	n/a	0.1114	1.748	-0.147	-0.366 – 0.071	0.186
	Examination number	n/a	n/a	0.0011	1.204	-0.001	-0.003 – 0.001	0.273
	Breed-estimated bodyweight (kg)	n/a	n/a	0.0870	17.229	0.361	0.191 – 0.532	<0.001*
	Age (years)	n/a	n/a	0.0577	5.077	-0.130	-0.243 – -0.017	0.024[FDR]
Any and increasing reactivity to intra-oral examination (n=430)	Examiner	MB	MJ	0.1738	20.875	0.794	0.453 – 1.134	<0.001*
	Sex	Female	Male	0.1548	9.136	0.468	0.165 – 0.771	0.003*
	Examination location (show or stud)	Show	Stud	0.2318	6.583	0.595	0.140 – 1.049	0.010*
	Ear type	Erect	Lop	0.2607	0.572	-0.197	-0.708 – 0.314	0.449
	Fur length[a]	Overall model	n/a	n/a	2.692	n/a	n/a	0.442
	Head shape	n/a	n/a	0.1210	0.455	0.082	-0.156 – 0.319	0.500
	Examination number	n/a	n/a	0.0008	0.186	0.000	-0.001 – 0.002	0.666
	Breed-estimated bodyweight (kg)	n/a	n/a	0.1222	9.914	0.385	0.145 – 0.624	0.002*
	Age (years)	n/a	n/a	0.0704	1.910	-0.097	-0.235 – 0.041	0.167

* Indicates significant p-value at the false discovery rate corrected threshold of p<0.019.

[FDR] indicates nonsignificant trends reported for completeness, after controlling for the false discovery rate.

[a] This variable was not significant at the overall model level, so the post-hoc pairwise comparisons are not reported.
